# Terrestrial arthropods of Steel Creek, Buffalo National River, Arkansas. II. Sawflies (Insecta: Hymenoptera: "Symphyta")

**DOI:** 10.3897/BDJ.4.e8830

**Published:** 2016-05-09

**Authors:** Michael Joseph Skvarla, David R. Smith, Danielle M. Fisher, Ashley P.G. Dowling

**Affiliations:** ‡ University of Maryland, University Park, Maryland, United States of America; § University of Arkansas, Fayetteville, Arkansas, United States of America; | Systematic Entomology Laboratory, Agricultural Research Service, U. S. Department of Agriculture, c/o National Museum of Natural History, Smithsonian Institution, Washington, D.C., United States of America

**Keywords:** Hymenoptera, "Symphyta", Argidae, Cimbicidae, Diprionidae, Orussidae, Pamphiliidae, Pergidae, Siricidae, Tenthredinidae, Xyelidae, Xiphydriidae, state record, range expansion, Interior Highlands, Boston Mountains

## Abstract

**Background:**

This is the second in a series of papers detailing the terrestrial arthropods collected during an intensive survey of a site near Steel Creek campground along the Buffalo National River in Arkansas. The survey was conducted over a period of eight and a half months using twelve trap types – Malaise traps, canopy traps (upper and lower collector), Lindgren multifunnel traps (black, green, and purple), pan traps (blue, purple, red, white, and yellow), and pitfall traps – and Berlese-Tullgren extraction of leaf litter.

**New information:**

We provide collection records for 47 species of "Symphyta" (Insecta: Hymenoptera), 30 of which are new state records for Arkansas: (Argidae) *Sterictiphora serotina*; (Cimbicidae) *Abia americana*; (Diprionidae) *Monoctenus fulvus*; (Orussidae) *Orussus terminalis*; (Pamphiliidae) *Onycholyda luteicornis*, *Pamphilius ocreatus*, *P. persicum*, *P. rileyi*; (Pergidae) *Acordulecera dorsalis, A. mellina, A. pellucida*; (Tenthredinidae) *Caliroa quercuscoccineae*, *Empria coryli*, *Hoplocampa marlatti*, *Macrophya cassandra*, *Monophadnoides conspiculatus, Monophadnus bakeri, Nematus abbotii, Neopareophora litura, Pachynematus corniger, Paracharactus rudis, Periclista marginicollis, Pristiphora banski, P. chlorea, Strongylogaster impressata, S. remota, Taxonus epicera, Thrinax albidopictus, T. multicinctus, Zaschizonyx montana*; (Xiphydriidae) *Xiphydria tibialis*.

## Introduction

The Interior Highlands is a mountainous physiogeographic region in the central United States that has remained unsubmerged and unglaciated since it was upthrust 270 mya (Bretz 1965, Robison and Allen 1995, Guccione 2008, U.S. Geological Survey 2014). It has acted as a refugium during times of inhospitable climate and as a result is a region of high biodiversity and endemism (Skvarla et al. 2015). However, terrestrial arthropods of the region have been understudied and undersurveyed compared to similar areas, such as the Southern Appalachains (Skvarla 2015). This paper, which focuses on "Symphyta", is the second in a series that intends to begin to rectify this situation (see Skvarla et al. 2015 for select Coleoptera).

"Symphyta", which are commonly referred to as sawflies and woodwasps because of the serrated or saw-shaped ovipositor present in many taxa and the habit of some taxa to bore into wood, is a paraphyletic grade of basal hymenopterans (Heraty et al. 2011). The majority of species are phytophagous, feeding both externally on leaves and internally as leaf miners and wood- and stem-borers on both monocots and dicots, though Orussidae, which are parasitic on wood-boring Hymenoptera and Coleoptera, are the notable exception. Historically, relatively few local or regional surveys have been reported in North America north of Mexico, though a number of studies conducted in eastern states have been published in recent years (e.g., Braud et al. 2003, Barrows and Smith 2014, Smith 2006b, Smith 2008c, Smith 2008a).

Herein we provide collection records for 47 species of sawflies collected in Arkansas.

## Sampling methods

### Sampling description

The sampling protocol was covered in detail by Skvarla et al. 2015. The following summary is provided for convenience.

The following traps were maintained within a 4 ha site at Steel Creek, Buffalo National River, Arkansas (see Geographic coverage for a description of the site): five Malaise traps, twenty-five pan traps (five of each color: blue, purple, red, yellow, white); fifteen Lindgren multi-funnel traps (five of each color: black, green, purple); four SLAM (Sea, Land, and Air Malaise) traps with top and bottom collectors placed in or just below the canopy; and seventeen pitfall trap sets. Additionally, ten leaf litter samples were collected for Berlese extraction when traps were serviced.

Trap placement began on 8 March 2013 and all traps were set by 13 March 2013, except Lindgren funnels, which were set on 1 April 2013. Traps set earlier than 13 March were reset on that date in order to standardize trap catch between traps. Traps were serviced approximately every two weeks. The last collection of pitfall traps and pan traps occurred on 6 November 2013; Malaise, SLAM, and Lindgren funnel traps were run for an additional month, with the final collection on 4 December 2013. In total, 1311 samples were collected.

Propylene glycol (in the form of Peak RV & Marine Antifreeze) was used as the preservative in all traps as it is non-toxic, inexpensive, and preserves specimens reasonably well (Skvarla et al. 2014). Insect escape was impeded by the addition of unscented, hypoallergenic dish detergent to the propylene glycol to act as a surfactant. Trap catch was sieved in the field and stored in Whirl-Pak bags in 90% ethanol until sorting.

### Quality control

Samples were coarse-sorted to suborder using a Leica MZ16 stereomicroscope illuminated with a Leica KL1500 LCD light source and a Wild M38 stereomicroscope illuminated with an Applied Scientific Devices Corp. Eco-light 20 fiber optic light source. After sorting, specimens were stored in 2 mL microtubes in 70% ethanol.

Specimens were identified by author Smith, who is an internationally recognized sawfly expert. Exemplar specimens are deposited in the National Museum of Natural History, Smithsonian Institution (USNM), with the remainder deposited in the University of Arkansas Arthropod Museum (UAAM). Distribution information is primarily from Smith 1979a; additional distribution references are indicated by species in "Notes on Select Species" below.

## Geographic coverage

### Description

The survey was conducted within a 4 hectare plot established at Steel Creek along the Buffalo National River in Newton County, Arkansas. The site is primarily 80–100 year old mature second-growth Eastern mixed deciduous forest dominated by oak (*Quercus*) and hickory (*Carya*), though American beech (*Fagus grandifolia*) and eastern red cedar (*Juniperus virginiana*) are also abundant. A glade (10 m x 30 m) with sparse grasses was present within the boundaries of the site.

### Coordinates

36.0367 and 36.0397 Latitude; -93.3917 and -93.3397 Longitude.

## Usage rights

### Use license

Creative Commons CCZero

## Data resources

### Data package title

Steel Creek survey

### Number of data sets

1

### Data set 1.

#### Data set name

Steel Creek Symphyta - DarwinCore

#### Data format

Darwin Core Archive

#### Number of columns

34

#### Download URL

http://dx.doi.org/10.5061/dryad.5rc70

#### 

**Data set 1.** 

Column label	Column description
typeStatus	Nomenclatural type applied to the record
catalogNumber	Unique within-project and within-lab number applied to the record
recordedBy	Who recorded the record information
individualCount	The number of specimens contained within the record
lifeStage	Life stage of the specimens contained within the record
kingdom	Kingdom name
phylum	Phylum name
class	Class name
order	Order name
family	Family name
genus	Genus name
specificEpithet	Specific epithet
scientificNameAuthorship	Name of the author of the lowest taxon rank included in the record
scientificName	Complete scientific name including author and year
taxonRank	Lowest taxonomic rank of the record
country	Country in which the record was collected
countryCode	Two-letter country code
stateProvince	State in which the record was collected
county	County in which the record was collected
municipality	Closest municipality to where the record was collected
locality	Description of the specific locality where the record was collected
verbatimElevation	Average elevation of the field site in meters
verbatimCoordinates	Approximate center point coordinates of the field site in GPS coordinates
verbatiumLatitude	Approximate center point latitude of the field site in GPS coordinates
verbatimLongitude	Approximate center point longitude of the field site in GPS coordinate
decimalLatitude	Approximate center point latitude of the field site in decimal degrees
decimalLongitude	Approximate center point longitude of the field site in decimal degrees
georeferenceProtocol	Protocol by which the coordinates were taken
identifiedBy	Who identified the record
eventDate	Date or date range the record was collected
habitat	Description of the habitat
language	Two-letter abbreviation of the language in which the data and labels are recorded
institutionCode	Name of the institution where the specimens are deposited
basisofRecord	The specific nature of the record

## Additional information

### Results

We collected and identified 468 specimens representing 47 species and 31 genera during this study (Table 1). Thirty-one species (including *Orussus minutus*, which was recently reported from Arkansas by Skvarla et al. 2015), which represent 66% of the total species collected, are newly recorded in Arkansas.

All trap types except Berlese extraction of leaf litter produced sawflies. While an in in-depth analysis of the data is in preparation, we include here the total number of species and specimens collected per trap type and the average number of specimens collected per trap (total specimens per trap type/total number traps per trap type) (Table 2). Malaise traps collected the highest number of species, while green Lindgren funnel traps collected the highest average number of specimens per trap.

### Known ranges and hosts of newly reported species

*Sterictiphora serotina* (Argidae) (**new state record**) has been reported from Maine, Massachusetts, Connecticut, New York, Maryland, New Jersey, Pennsylvania, Virginia, and Tennessee (Smith 1969a). Dyar 1897 and Eiseman 2015 described larval feeding patterns on *Prunus*.

*Abia americana* (Cimbicidae) (**new state record**) is widespread throughout North America and has been reported from Quebec, Connecticut, and Ontario, west through Manitoba, Iowa, Alaska, New Mexico, and California (Britton 1925, Middlekauff 1956, Furniss 1972, Still et al. 1974). Larvae feed on honeysuckle (*Lonicera*), including non-native species (Britton 1925, Middlekauff 1956, Baron and Bisdee 1984).

*Monoctenus fulvus* (Diprionidae) (**new state record**) has been recorded from Virginia, West Virginia, Kansas, Oklahoma, and Texas (Marlatt 1888, Smith et al. 2010). Marlatt 1888 provided a life history account of the species attacking red cedar (*Juniperus virginiana*).

The *Orussus minutus* (Orussidae) specimens collected during this study were previously reported as new state records by Skvarla et al. 2015. The species was previously known from New York south to Georgia, west to Illinois. Though the larvae of *O. minutus* are unknown, all other orussids are parasitioids upon wood-boring Coleoptera and Hymenoptera larvae and *O. minutus* likely do the same.

*Orussus terminalis* (Orussidae) (**new state record**) occurs from New England and Ontario west to Iowa and Illinois (Middlekauff 1983).

*Onycholyda luteicornis* (Pamphiliidae) (**new state record**) occurs from Nova Scotia and New Brunswick south to Maryland, Virginia, and Florida, west to Tennessee and Illinois (Middlekauff 1964). Larvae feed on leaf folds of *Rubus* (Smith 2008c).

*Pamphilius ocreatus* (Pamphiliidae) (**new state record**) occurs from Connecticut south to Virginia, west to Indiana (Shinohara and Smith 1983). Dyar 1895 reported the larvae are web spinners that roll leaves on hazel (*Corylus rostrata*).

*Pamphilius persicum* (Pamphiliidae) (**new state record**) is widespread in eastern North America and has been reported from Quebec and Connecticut south through Pennsylvania, New Jersey, Virginia and Florida, west to Tennessee, Minnesota, Nebraska, and Texas (Walden 1907a, Middlekauff 1964, Shinohara 1985). Outbreaks in which larvae partially defoliate peach trees (*Prunus persica* (L.) Batsch 1801 nec Stokes 1812 nor (L.) Siebold & Zucc. 1845) and hundreds of adults can be collected via sweep netting within a few seconds have been recorded (Walden 1907b, Walden 1907a, Walden 1908a, Walden 1908b)

*Pamphilius rileyi* (Pamphiliidae) (**new state record**) occurs from Connecticut south to Virginia and West Virginia, west to Michigan, Iowa, and Missouri (Middlekauff 1964, Shinohara 1985). The larval food host has been reported to be *Amelanchier*, though Shinohara 1985 indicated that this is based off a single male specimen that was reported to be ovipositing and so likely in error.

*Acordulecera dorsalis*, *A. mellina*, and *A. pellucida* (Pergidae) (**new state records**) are widespread in eastern North America from Quebec, Maryland, Virginia, and Georgia, west to Oklahoma and Texas (Smith 1979a). *Acordulecera dorsalis* has been reported from *Quercus*, *Carya*, *Juglans*, and *Castanea*, while the larval food plants of *A. mellina* and *A. pellucida* are unknown (Smith 2008c).

*Caliroa quercuscoccineae* (Tenthredinidae) (**new state record**), commonly known as the scarlet oak sawfly, is widespread in eastern North America from Maine south to North Carolina, west to Minnesota, Illinois, Missouri, and Louisiana (Smith 1979a, Nordin and Johnson 1983, Boggs et al. 1999). Though the species is native, outbreaks that cause significant damage to large areas (up to 1.5 million acres) have been recorded (Matuszewski and Barry 1975, Matuszewski 1976, Matuszewski and Ward 1977, Nordin and Johnson 1983).

*Empria coryli* (Tenthredinidae) (**new state record**) occurs from New Hampshire and New York, west to Wisconsin, Iowa, and Missouri (Smith 1979c). Larvae have been recorded from hazel (*Corylus*) (Smith 1979a, Smith 2006b).

*Hoplocampa marlatti* (Tenthredinidae) (**new state record**) occurs from Maine south to Georgia, west to Iowa, Kansas, and Colorado (Rohwer 1918, Ross 1943, Smith 1979b, Braud et al. 2003, Smith 2006a, Smith 2008a, Barrows and Smith 2014). Larvae have been reported to feed on *Prunus* (Smith 2008a).

*Macrophya cassandra* (Tenthredinidae) (**new state record**) is known from Nova Scotia south to Georgia, west to Manitoba, South Dakota, and Montana (Gibson 1980). Larvae have been reported to feed on *Carya* (Smith 2006b).

*Monophadnoides conspiculatus* (Tenthredinidae) (**new state record**) occurs from Nova Scotia and Ontario, south to North Carolina and Tennessee (Smith 1969a).

*Monophadnoides pauper* (Tenthredinidae) (**new state record**) occurs from Labrador and Maryland west to Missouri, Kansas, Colorado, and Alberta (Smith 1969a).

*Monophadnus bakeri* (Tenthredinidae) (**new state record**) is recorded from Maryland, Virginia, Tennessee, West Virginia, Kansas, and Montana (Smith 1969a).

*Nematus abbotii* (Tenthredinidae) (**new state record**) is widespread in eastern North America and has been recorded from Ontario south to Alabama and Georgia, west to Minnesota, Kansas, Iowa, and Missouri (Smith 2008b). It has been reported to feed on black locust (*Robinia pseudoacacia* L.) and is suspected of being univoltine as adults have only been collected during the spring, but little else is known about the species' biology (Smith 2008b).

*Neopareophora litura* (Tenthredinidae) (**new state record**), commonly called the blueberry sawfly, has been recorded from New Brunswick and Newfoundland south to Virginia and west to Illinois. It feeds on *Vaccinium* L., especially low-bush blueberry (*V. angustifolium*) on which it is occasionally a minor pest, so is likely present wherever host plants are found (Neilson 1955, Neilson 1958, Smith 1979b, Collins et al. 1994).

*Pachynematus corniger* (Tenthredinidae) (**new state record**) occurs from Newfoundland and Quebec south to Georgia, west to Minnesota, Illinois and Kansas; it has also been found in Colorado (Marlatt 1896, Crevecoeur 1922, Smith 1979b, Environment Canada 2014). Larval food plants have been reported to be *Carex* and possibly grasses (Smith 2006b, Smith 2008a).

*Paracharactus rudis* (Tenthredinidae) (**new state record**) occurs from Maine and Quebec south to Georgia, west to Saskatchewan and Colorado (Smith 1969b).

*Periclista marginicollis* (Tenthredinidae) (**new state record**), commonly known as the pecan sawfly, occurs from Ontario and Connecticut south to Florida, west to Iowa, Kansas, Oklahoma, and Texas. Host plants include species of *Carya* Nutt., including pecan (*C. illinoinensis* (Wangenh.) K.Koch), on which it can occasionally cause economic damage (Smith 1969a, Smith 1979a, Smith et al. 1996, Ree and Knutson 1997, Dickey and Medina 2010).

*Pristiphora banski* (Tenthredinidae) (**new state record**) occurs from New York and West Virginia south to North Carolina, as well as Wyoming, where is feeds on *Vaccinium* L. (Smith 1979b, Braud et al. 2003, Smith 2006b, Smith 2008c, Smith 2008a, Barrows and Smith 2014). It is likely the species will be found in North America wherever larval host plants are located.

*Pristiphora chlorea* (Tenthredinidae) (**new state record**) is widespread in North America and has been recorded from New Brunswick, south to Florida, west to Manitoba, Oregon, and Texas (Frost 1969, Smith 1979b, Smith 2006b, Smith 2008c, Smith 2008a, Barrows and Smith 2014). Larvae feed on *Quercus* (Smith 2006b).

*Strongylogaster impressata* (Tenthredinidae) (**new state record**) is widespread in North America and has been recorded from New Brunswick and Quebec, south to North Carolina, west to Minnesota, Colorado, and New Mexico. It feeds on ferns, including *Pteridium aquilinum*, though it has been shown that cyanogenic compounds within some ferns impair feeding and development (Smith 1969b, Schreiner et al. 1984).

*Strongylogaster remota* (Tenthredinidae) (**new state record**) is an uncommonly collected species known from Quebec, Maryland, Virginia, Pennsylvania, and Tennessee (Smith 1969b, Barrows and Smith 2014, Smith 1979b, Smith 2006b, Smith 2008c, Smith 2008a).

*Taxonus epicera* (Tenthredinidae) (**new state record**) occurs in eastern North America from New Hampshire and Ontario west to Kansas and Texas (Smith 1979a, Smith 1979b).

*Thrinax albidopicta* (Tenthredinidae) (**new state record**) occurs from Nova Scotia and New Brunswick, south to Alabama, and west to Minnesota, Iowa, and Kansas (Rohwer 1910, Viereck 1916, Smith 1979b, Smith 2006b, Smith 2008a). It has historically been placed in *Hemitaxonus*, which was recently synonymized with *Thrinax* by Blank 2002. Larvae have been recorded on the ferns *Onoclea sensibilis* and *Osmunda* (Smith 1969c). Larval host plants are ferns (Smith 2008a).

*Thrinax multicinctus* (Tenthredinidae) (**new state record**) has been recorded from Maine, Ontario, Maryland, Ohio, and Tennessee (Smith 1969b, Smith 1979b, Smith 2008a). Similarly to *Thrinax albidopicta*, Blank 2002 transferred *T. multicinctus* from *Hemitaxonus* to *Thrinax*. Larvae have been reported from lady-ferns (*Athyrium*).

*Zaschizonyx montana* (Tenthredinidae) (**new state record**) is widespread in North America and occurs from Ontario, Minnesota, Illinois, and Missouri west to British Columbia, Washington, and California (Smith 1979c).

*Xiphydria tibialis* (Xiphydriidae) (**new state record**) is widespread in eastern North America and occurs from Nova Scotia and Quebec south to Florida, west to Wisconsin, Illinois, and Kansas (Smith 1976). Larvae bore in small limbs of a number of trees, including *Acer*, *Betula*, *Fagus*, *Malus*, *Quercus*, *Tilia*, and *Ulmus* (Smith 2008c).

### Discussion

Thirty-one species are recorded as new to Arkansas. The majority (23 species) are wide-ranging species found throughout North America east of the Rocky Mountains and many have been collected in states bordering Arkansas. While it is unsurprising that they also occur in Arkansas, they serve to highlight how little collecting and survey work some groups have historically received in the state.

Specimens of eight species – *Monophadnoides conspiculatus, Neopareophora litura, Orussus minutus, O. terminalis*, *Pamphilius ocreatus*, *P. rileyi, Strongylogaster remota*, and *Thrinax multicinctus* – represent significant western range extensions of hundreds of kilometers from previously known localities. However, it is likely that they do not represent disjunct populations confined to the Interior Highlands and that increased sampling effort in eastern North America will uncover additional specimens from new localities.

Malaise traps collected the highest number of species (28). Green Lindgren funnel traps collected the second highest number of species (17), which while fewer than Malaise traps is higher than all other trap types; they also collected slightly more specimens per trap than Malaise traps. Most species were represented by 10 or fewer specimens, while only four species were represented by 25 or more specimens.

## Figures and Tables

**Table 1.** Species collected, including total number of specimens. New state records are indicated by an asterisk (*).

**Family**	**Genus**	**Species**	**Number of specimens**
Argidae	* Arge *	*Arge humeralis* (Palisot de Beauvois, 1809)	1
Argidae	* Arge *	*Arge macleayi* (Leach, 1817)	1
Argidae	* Sterictiphora *	*Sterictiphora serotina* Smith, 1969 *	2
Cimbididae	* Abia *	*Abia americana* (Cresson, 1880)*	2
Diprionidae	* Monoctenus *	*Monoctenus fulvus* (Norton, 1872)*	9
Orussidae	* Orussus *	*Orussus minutus* Middlekauff, 1983*	2
Orussidae	* Orussus *	*Orussus terminalis* Newman, 1838*	1
Pamphiliidae	* Onycholyda *	*Onycholyda luteicornis* (Norton, 1869)*	3
Pamphiliidae	* Pamphilius *	*Pamphilius ocreatus* (Say, 1836)*	3
Pamphiliidae	* Pamphilius *	*Pamphilius persicum* MacGillivray, 1907*	1
Pamphiliidae	* Pamphilius *	*Pamphilius rileyi* (Cresson, 1880)*	2
Pergidae	* Acordulecera *	*Acordulecera dorsalis* Say, 1836*	209
Pergidae	* Acordulecera *	*Acordulecera mellina* MacGillivray, 1908*	1
Pergidae	* Acordulecera *	*Acordulecera pellucida* (Konow, 1898)*	18
Siricidae	* Tremex *	*Tremex columba* (Linnaeus, 1763)	2
Tenthredinidae	* Caliroa *	*Caliroa quercuscoccineae* (Dyar, 1894)*	2
Tenthredinidae	* Craterocercus *	*Craterocercus obtusus* (Klug, 1816)	1
Tenthredinidae	* Dolerus *	*Dolerus neoagcistus* MacGillivray, 1923	1
Tenthredinidae	* Empria *	*Empria coryli* (Dyar, 1897)*	5
Tenthredinidae	* Empria *	*Empria maculata* (Norton 1861)	6
Tenthredinidae	* Eupareophora *	*Eupareophora parca* (Cresson, 1880)	36
Tenthredinidae	* Hoplocampa *	Hoplocampa marlatti*	6
Tenthredinidae	* Macrophya *	*Macrophya cassandra* Rohwer, 1911*	2
Tenthredinidae	* Macrophya *	*Macrophya formosa* (Klug, 1817)	1
Tenthredinidae	* Macrophya *	*Macrophya macgillivrayi* Gibson, 1980	2
Tenthredinidae	* Macrophya *	*Macrophya pulchella* (Klug, 1817)	3
Tenthredinidae	* Monophadnoides *	*Monophadnoides conspiculatus* MacGillivray, 1908*	1
Tenthredinidae	* Monophadnoides *	*Monophadnoides pauper* (Provancher, 1882)*	5
Tenthredinidae	* Monophadnoides *	*Monophadnoides rubi* (Harris, 1845)	2
Tenthredinidae	* Monophadnus *	*Monophadnus bakeri* Smith, 1969*	1
Tenthredinidae	* Nefusa *	*Nefusa ambigua* (Norton, 1867)	2
Tenthredinidae	* Nematus *	*Nematus abbotii* (W.F. Kirby, 1882)*	1
Tenthredinidae	* Nematus *	*Nematus tibialis* Newman, 1837	1
Tenthredinidae	* Neopareophora *	*Neopareophora litura* (Klug, 1816)*	15
Tenthredinidae	* Pachynematus *	*Pachynematus corniger* (Norton, 1861)*	1
Tenthredinidae	* Paracharactus *	*Paracharactus rudis* (Norton, 1861)*	54
Tenthredinidae	* Periclista *	*Periclista marginicollis* (Norton, 1861)*	25
Tenthredinidae	* Pristiphora *	*Pristiphora banski* Marlett, 1896*	10
Tenthredinidae	* Pristiphora *	*Pristiphora chlorea* (Norton, 1867)*	2
Tenthredinidae	* Strongylogaster *	*Strongylogaster impressata* Provancher, 1878*	7
Tenthredinidae	* Strongylogaster *	*Strongylogaster remota* Rohwer, 1912*	1
Tenthredinidae	* Taxonus *	*Taxonus epicera* (Say, 1836)*	1
Tenthredinidae	* Thrinax *	*Thrinax albidopicta* (Norton, 1868)*	7
Tenthredinidae	* Thrinax *	*Thrinax multicinctus* (Hall, 1918)*	1
Tenthredinidae	* Zaschizonyx *	*Zaschizonyx montana* (Cresson, 1865)*	5
Xyelidae	* Xyela *	*Xyela pini* Rohwer, 1913	3
Xiphydriidae	* Xiphydria *	*Xiphydria tibialis* Say, 1824*	1

**Table 2.** Number of species and specimens collected per trap type

**Trap type**	**Total species**	**Total specimens**	**Average specimens per trap**
Malaise trap	28	181	36.20
Lindgren funnel, green	17	165	41.25
Lindgren funnel, purple	6	9	2.25
Lindgren funnel, black	3	5	1.25
Pan trap, yellow	8	29	5.80
Pan trap, blue	6	19	3.80
Pan trap, red	8	16	3.20
Pan trap, white	5	17	3.40
Pan trap, purple	3	11	2.20
SLAM trap, lower	3	5	1.25
SLAM trap, upper	1	2	0.50
Pitfall	1	4	0.24
Berlese	0	0	0
